# The next frontier in antibody-drug conjugates: challenges and opportunities in cancer and autoimmune therapy

**DOI:** 10.20517/cdr.2025.49

**Published:** 2025-07-03

**Authors:** Meijiang Zhou, Zhiwen Huang, Zijun Ma, Jun Chen, Shunping Lin, Xuwei Yang, Quan Gong, Zachary Braunstein, Yingying Wei, Xiaoquan Rao, Jixin Zhong

**Affiliations:** ^1^Division of Rheumatology and Immunology, Department of Internal Medicine, Tongji Hospital, Tongji Medical College, Huazhong University of Science and Technology, Wuhan 430030, Hubei, China.; ^2^Sinopharm Dongfeng General Hospital (Hubei Clinical Research Center of Hypertension), Hubei Key Laboratory of Wudang Local Chinese Medicine Research, Hubei University of Medicine, Shiyan 442000, Hubei, China.; ^3^Department of Rheumatology, Fujian Medical University Union Hospital, Fuzhou 350001, Fujian, China.; ^4^Fujian Institute of Clinical Immunology, Fuzhou 350001, Fujian, China.; ^5^Department of Immunology, School of Medicine, Yangtze University, Jingzhou 434023, Hubei, China.; ^6^Division of Hematology, Department of Internal Medicine, James Comprehensive Cancer Center, The Ohio State University Wexner Medical Center, Columbus, OH 43210, USA.; ^7^Division of Cardiology, Department of Internal Medicine, Tongji Hospital, Tongji Medical College, Huazhong University of Science and Technology, Wuhan 430030, Hubei, China.; ^8^Key Laboratory of Vascular Aging (HUST), Ministry of Education, Wuhan 430030, Hubei, China.

**Keywords:** Drug resistance, ADC design innovations, combination therapies with ADCs

## Abstract

Antibody-Drug Conjugates (ADCs) have achieved significant success in cancer therapy by combining the targeting specificity of monoclonal antibodies with cytotoxic payloads. However, the concomitant issue of drug resistance has become increasingly prominent, with primary mechanisms including alterations in target antigen expression, impaired drug transport, and inhibition of cell death pathways. ADCs have also shown emerging therapeutic potential in the treatment of autoimmune diseases; for instance, ABBV-3373 has achieved initial success in this area, yet it also faces unique challenges such as the safety of long-term administration, immunogenicity, and heterogeneity of target cells. Addressing these challenges requires multidimensional innovations, including optimizing molecular design, exploring combination therapy strategies, and introducing artificial intelligence (AI)-assisted development. These efforts aim to transition ADCs from the traditional “targeted killing” paradigm to intelligent and personalized precision delivery systems, thereby offering more therapeutic options for patients with cancer and autoimmune diseases.

## INTRODUCTION

**Figure 1:**
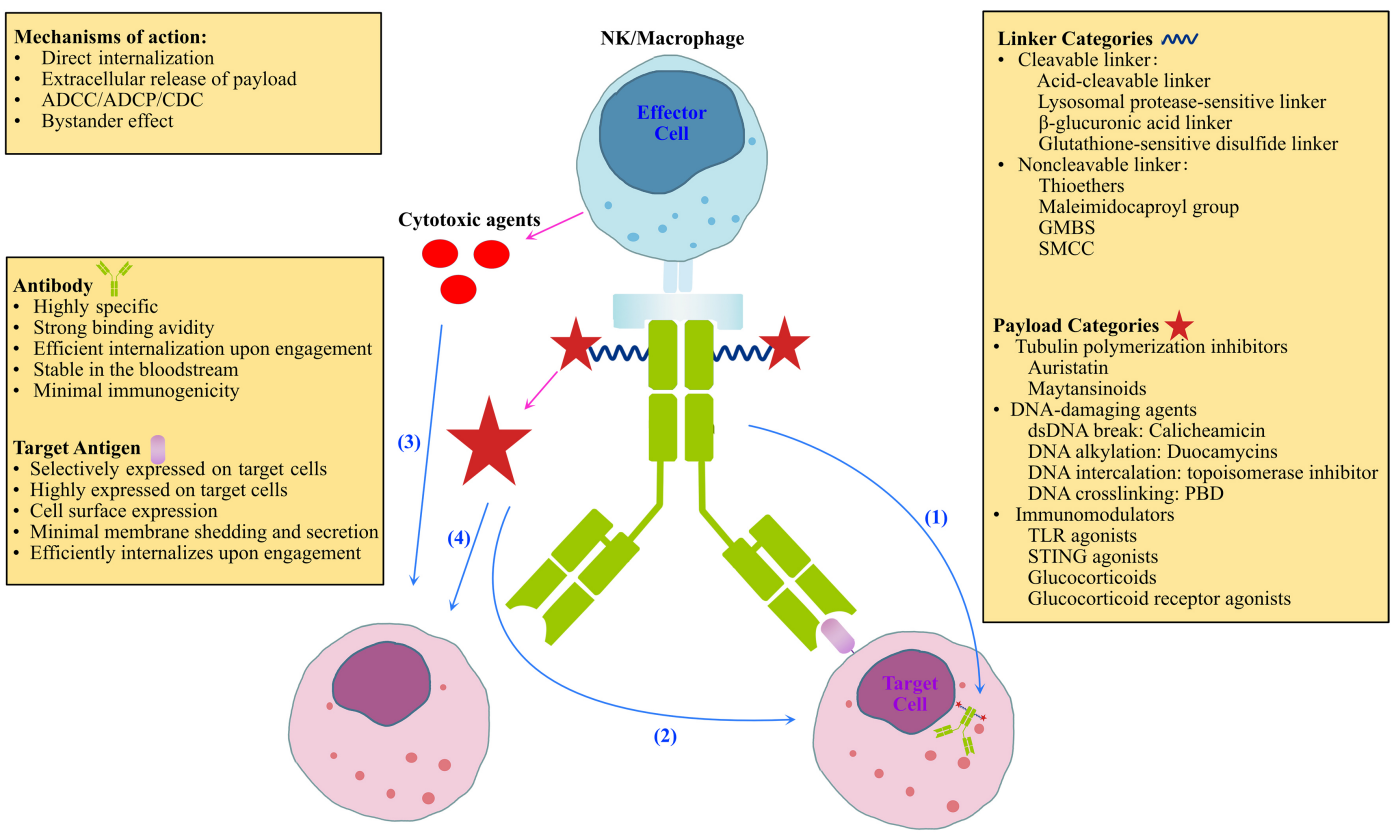
ADC molecular structure and mechanisms of action. ADCs contain three key components: antibody, linker, and payload. They exert their function through four primary mechanisms of action: (1) Direct Internalization: The ADC antibody binds to the target antigen on the cell surface, leading to the internalization of the entire complex into the target cell; (2) Extracellular Release of Payload: The payload is released outside the cell, directly exerting cytotoxic effects on the target cells; (3) ADCC/ADCP/CDC (Antibody-Dependent Cellular Cytotoxicity/Phagocytosis/CDC): By binding to the Fc fragment of the ADC antibody, effector cells (e.g., NK cells or macrophages) are recruited to destroy target cells via immune-mediated mechanisms, including cell killing, phagocytosis, and complement activation; (4) Bystander Effect: The payload, once released, can diffuse to adjacent cells, effectively killing neighboring cells even if they do not express the target antigen. ADC: Antibody-drug conjugate; ADCC: antibody-dependent cellular cytotoxicity; ADCP: antibody-dependent cellular phagocytosis; CDC: complement-dependent cytotoxicity; Fc: fragment crystallizable; GMBS: N-γ-maleimidobutyryl-oxysuccinimide ester; NK: natural killer; PBD: pyrrolobenzodiazepine; SMCC: succinimidyl-4-(N-maleimidomethyl)cyclohexane-1-carboxylate; STING: stimulator of interferon genes; TLR: toll-like receptor.

By combining the precise tumor-targeting properties of antibodies with the powerful cytotoxic effects of their drug payloads, antibody-drug conjugates (ADCs) hold great promise in cancer treatment and are often conceptualized as the “magic bullets” of cancer therapy^[1]^. The ADC is a composite therapeutic comprising three key components: a monoclonal antibody, a linker, and a payload [Figure 1]. By integrating the advantages of highly specific targeting and potent cytotoxicity, ADCs allow for precise and efficient elimination of target cells while minimizing off-target effects on normal tissues, thereby optimizing therapeutic outcomes. Through the recognition of unique antigens (e.g., tumor-specific antigens) present on target cells or tissues, ADCs can deliver drugs with potent cytotoxic or immunomodulatory activity, offering favorable bioavailability and relatively lower adverse reactions^[2]^. In the decades since their development, ADCs have primarily been deployed in oncology^[3,4]^. However, advances in ADC technology and growing clinical demands have broadened its scope beyond cancer. Autoimmune diseases, which share key immunological pathways with malignancies, have emerged as a natural frontier for ADC therapies, with promising early results already reported^[2,5]^. This review first explores the overlapping biology of tumors and autoimmune disorders, and then delves into the design principles and evolution of ADC platforms. Subsequently, we will outline their current applications in both oncology and autoimmune disease treatment, followed by a discussion of persistent challenges such as toxicity and drug resistance. Finally, we discuss future opportunities, particularly in leveraging AI to optimize drug targeting and combination strategies.

## RELATIONSHIP BETWEEN CANCER AND AUTOIMMUNE DISEASES

Immunologically, cancer and autoimmune diseases both involve dysregulated immune responses, though through distinct mechanisms. In cancer, tumors actively create an immunosuppressive microenvironment, although localized inflammatory responses may coexist with cancer-driven immunosuppression in certain circumstances. This involves mechanisms such as upregulating checkpoint proteins (e.g., PD-L1), recruiting regulatory T cells (Tregs), and secreting cytokines (e.g., IL-10, TGF-β) that suppress antitumor immunity^[5-8]^. These strategies allow tumors to escape destruction and promote growth. In autoimmune diseases, in contrast, the core issue is a breakdown of self-tolerance, where the immune system mistakenly attacks self-antigens. This involves overactivation of inflammatory pathways [e.g., IFN-α in lupus, TNF-α in rheumatoid arthritis (RA)] and dysregulated B/T cell responses, leading to chronic inflammation and progressive tissue damage. Unlike the immune suppression seen in cancer, autoimmune conditions are driven by immune hyperactivation, though both scenarios result from failed immune regulation^[5-8]^.

Despite these opposing immune states, a common thread unites both pathologies: the central role of a chronic inflammatory microenvironment. The development of both tumors and autoimmune diseases relies on the establishment and progression of a chronic inflammatory microenvironment, and they involve largely similar signaling pathways^[5,7,9]^. In both types, chronic inflammation triggers disease progression through the release of large quantities of pro-inflammatory cytokines (e.g., TNFα, IL-6, IL-1β), which in turn activate core signaling pathways such as STAT3 and PI3K. This inflammatory microenvironment regulates the function of immune cells in a manner that, in cancer, fosters genomic instability, angiogenesis, and immune escape of tumor cells, whereas in autoimmune diseases, it results in persistent attacks on self-antigens and leads to extensive tissue damage^[5,7,9,10]^. Macrophages also play a pivotal role in the inflammatory settings of both disorders; imbalances in the M1/M2 macrophage ratio and function profoundly affect disease progression^[5]^. Furthermore, tissue hypoxia is an important factor in maintaining inflammation in both cancers and autoimmune diseases. The upregulation of Hypoxia-Inducible Factor 1α (HIF-1α) not only promotes angiogenesis but exacerbates tissue damage and immune dysregulation by modulating cellular metabolism and forming an acidic microenvironment. These shared features highlight that chronic inflammation, despite manifesting differently, serves as a crucial driving force in both cancer and autoimmune diseases^[5,7,11]^.

Moreover, the two disease forms exhibit parallels in immune checkpoint dysregulation. Immune checkpoints are typically used to modulate immune responses and maintain immune homeostasis. In cancer, tumor cells often express checkpoint ligands such as PD-L1, inhibiting T cell activity and helping the tumor evade the immune system. In autoimmune diseases, however, dysfunction of these same checkpoints can lead to excessive responses against self-antigens. In both conditions, altered expression and function of immune checkpoints can accelerate disease pathology. Therefore, immune checkpoints constitute a vital biological link between cancer and autoimmune diseases and represent attractive therapeutic targets for future treatments^[8,12,13]^.

Particularly noteworthy, hematologic malignancies and autoimmune diseases share a number of common molecular targets. For instance, CD19 is highly expressed in B cell malignancies [such as B cell lymphoma and acute lymphoblastic leukemia (ALL)] as well as in autoimmune disorders characterized by aberrant B cell activity, including systemic lupus erythematosus (SLE) and RA. Anti-CD19 CAR-T cell therapy and anti-CD19 monoclonal antibody therapies have already demonstrated efficacy in treating hematologic cancers, and more recently, CD19-targeted CAR-T cell therapy has been extended to autoimmune diseases^[14,15]^. A combination of anti-CD19 CAR-T cells and mycophenolate mofetil successfully induced both clinical and serological remission in a progressive anti-synthetase syndrome patient who was refractory to all existing treatments - including rituximab and azathioprine^[16,17]^. Another key antigen, the B cell maturation antigen (BCMA), is a specific marker of mature plasma cells that plays a role in both multiple myeloma (MM) and autoimmune diseases featuring disordered autoantibody production. In a clinical trial of bb2121 (an anti-BCMA CAR-T cell therapy) involving 128 patients with MM (NCT03361748), the objective response rate (ORR) reached 73%, demonstrating the considerable therapeutic potential of targeting BCMA in MM. Moreover, a study employing programmable phage immunoprecipitation sequencing (PhIP-Seq) to examine the proteome-wide autoreactome in different CAR-T cell therapies showed that anti-BCMA CAR-T therapy significantly reshaped the repertoire of autoantibodies, suggesting its potential importance in treating autoimmune conditions. These findings indicate that ADCs developed against hematologic malignancies could likely benefit patients with autoimmune diseases as well^[14,18-21]^.

## ADC THERAPY

The concept of ADC therapy was first proposed by Paul Ehrlich during his research on syphilis; he posited that linking chemotherapeutic drugs to antibodies could enhance therapeutic selectivity and efficacy. ADCs are composed of three core components: a specific monoclonal antibody, a cytotoxic payload, and a linker^[1]^. An ideal ADC should remain stable before reaching the target site and release the payload only at the target site under the guidance of the monoclonal antibody. The composition and interaction of these three key components will affect the specificity, potency, and ultimately the efficacy and toxicity of the ADC^[2,4,22,23]^. As of January 2025, the U.S. Food and Drug Administration (FDA) has approved 14 ADCs for clinical use, and over one hundred new ADC drugs are under development^[24-26]^. Table 1 lists ADCs approved by the FDA.

**Table 1:** ADCs approved by the FDA

**Targeted antigen**	**Antibody-drug conjugate**	**Linker**	**Linker type**	**Antibody type**	**Payload**	**Approved indications**
CD33	Gemtuzumab ozogamicin	Hydrazone	Cleavable	Humanized IgG1	N-acetyl-γ-calicheamicin	Acute myelogenous leukemia
CD30	Brentuximab vedotin	Dipeptide	Cleavable	Chimeric IgG1	MMAE	Hodgkin’s lymphoma
HER-2	Trastuzumab emtansine	Thioether	Noncleavable	Humanized IgG1	DM1	HER2 positive breast cancer
CD22	Inotuzumab ozogamicin	Hydrazone	Cleavable	Humanized IgG4	N-acetyl-γ-calicheamicin	r/rB cell acute lymphoblastic leukemia
CD22	Moxetumomab pasudotox	mc-VC-PABC	Cleavable	Chimeric with a stabilized Fv segment for fusion	PE38	r/r hair-cell leukemia
CD79b	Polatuzumab vedotin-piiq	mc-VC-PABC	Cleavable	Humanized IgG1	MMAE	r/r diffuse large B-cell lymphoma
Nectin-4	Enfortumab vedotin	mc-VC-PABC	Cleavable	Humanized IgG1	MMAE	Advanced urothelial carcinoma
HER-2	Trastuzumab deruxtecan	Tetrapeptide	Cleavable	Humanized IgG1	Dxd	Metastatic HER2-positive breast cancer
Trop-2	Sacituzumab govitecan	CL2A	Cleavable	Humanized IgG1	SN38	Triple-negative breast cancer
BCMA	Belantamab mafodotin	mc	Noncleavable	Humanized IgG1	MMAF	r/r multiple bone marrow cancer
CD19	Loncastuximab tesirine	Dipeptide	Cleavable	Humanized IgG1	PBD	r/r diffuse large B-cell lymphoma
Tissue factor	Tistotumab vedotin	mc-VC-PABC	Cleavable	Human IgG1	MMAE	Recurrent or metastatic cervical cancer
Folate receptor-α	Mirvetuximab soravtansine	sulfo-SPDB	Cleavable	Humanized IgG1	DM4	Ovarian cancer
Trop-2	Datopotamab deruxtecan	Tetrapeptide	Cleavable	Humanized IgG1	Dxd	Unresectable or metastatic, HR-positive, HER2-negative breast cancer

ADC: Antibody-drug conjugate; BCMA: B-cell maturation antigen; CD: cluster of differentiation; CL2A: cleavable linker 2A; DM1: derivative of maytansine 1; DM4: derivative of maytansine 4; Dxd: deruxtecan; Fv: variable fragment; HER2: human epidermal growth factor receptor 2; HR: hormone receptor; mc: maleimidocaproyl; mc-VC-PABC: maleimidocaproyl-valine-citrulline-para-aminobenzyl carbamate; MMAE: monomethyl auristatin E; MMAF: monomethyl auristatin F; PE38: pseudomonas exotoxin a fragment 38 kDa; PBD: pyrrolobenzodiazepine; r/r: relapsed or refractory; SN-38: 7-ethyl-10-hydroxycamptothecin; sulfo-SPDB: N-succinimidyl 4-(2-pyridyldithio)butanoate; Trop-2: trophoblast cell-surface antigen 2.

The monoclonal antibody, as the guiding core, needs to possess high target specificity and recognition capability, high internalization efficiency to trigger receptor-mediated endocytosis, and a long circulation half-life to improve bioavailability. To reduce immunogenicity, antibodies have evolved from early murine antibodies to humanized or even fully human antibodies^[26-28]^. The IgG1 subtype, due to its longer half-life and ability to trigger dual killing mechanisms, namely antibody-dependent cell-mediated cytotoxicity (ADCC), complement-dependent cytotoxicity (CDC) and antibody-dependent cellular phagocytosis (ADCP), has become the mainstream choice^[4,26,29]^.

The linker controls the timing and manner of payload release; it must remain stable in blood circulation while efficiently cleaving in the target environment^[4,29,30]^. Linkers are broadly categorized into two types: cleavable linkers, which include acid-sensitive linkers (e.g., the acylhydrazone linker in gemtuzumab ozogamicin), lysosomal protease-sensitive linkers [e.g., the valine-citrulline linker in brentuximab vedotin (BV)], reduction-sensitive linkers (dependent on glutathione cleavage), and β-glucuronide linkers (hydrolyzed by lysosomal β-glucuronidase)^[27,31-34]^. Non-cleavable linkers, such as succinimidyl-4-(N-maleimidomethyl)cyclohexane-1-carboxylate (SMCC), release the payload through metabolic degradation of the antibody, offering higher safety and a longer plasma half-life^[2]^. Table 2 lists common linker types with their release mechanism and representative ADCs.

**Table 2:** Common linker types with their release mechanism and representative ADCs

**Linker type**	**Cleavable**	**Cleavage/Release mechanism**	**Representative ADC**
Acid-Labile linkers	Yes	Hydrolyzed under acidic conditions (lysosomal pH ~4.8 or endosomal pH 5-6)	Gemtuzumab ozogamicin, inotuzumab ozogamicin
Lysosomal protease-sensitive linkers	Yes	Specific cleavage by lysosomal proteases (e.g., cathepsin B)	Brentuximab vedotin, loncastuximab tesirine
β-glucuronidase linkers	Yes	Cleaved by β-glucuronidase, which is highly expressed in lysosomes (hydrolyzing the glycosidic bond)	LCB14-0110 (not yet approved)
Reduction-sensitive linkers (glutathione-sensitive disulfide linker)	Yes	Relies on high intracellular GSH concentration in target cells for reducing the disulfide bond	Lorvotuzumab mertansine (not yet approved)
Noncleavable linker	No	Payload release occurs via degradation of the mAb in lysosomes	Trastuzumab emtansine, belantamab mafodotin

GSH: Glutathione; ADC: antibody-drug conjugate; mAb: monoclonal antibody.

The payloads must exhibit potent efficacy at very low concentrations and primarily include three categories^[35-37]^: (1) Tubulin Polymerization Inhibitors (e.g., auristatins such as MMAE and MMAF, and maytansinoid derivatives like DM), which induce apoptosis by blocking microtubule polymerization. It is particularly noteworthy that MMAE exhibits a significant bystander effect, meaning the released drug molecules can diffuse to neighboring tumor cells, thereby overcoming treatment limitations caused by tumor cell heterogeneity and enhancing overall therapeutic efficacy^[38-42]^; (2) DNA-damaging payloads, which can be subdivided into topoisomerase inhibitors (e.g., SN-38 and Dxd) that hinder DNA unwinding and exert a bystander effect^[43,44]^; DNA cross-linking agents (e.g., PBD dimers) that form covalent cross-links preventing DNA replication and DNA cleaving payloads (e.g., calicheamicin), which directly cleaves the DNA double helix^[26,40,45-48]^; (3) Immunomodulatory molecules, which achieve therapeutic effects by modulating immune responses^[49,50]^. Overall, the selection of these payloads requires balancing efficacy, safety, and pharmacokinetic properties to achieve the optimal therapeutic window for ADCs. Table 3 lists common payloads with their representative compounds and ADCs.

**Table 3:** Common payloads with their Representative compounds and ADCs

**Payload category**	**Mechanism of action**	**Bystander effect**	**Representative compounds**	**Representative ADCs**
Tubulin polymerization payloads	Inhibit tubulin polymerization, block mitosis	MMAE: strong MMAF: weaker DM1/DM4: moderate	Auristatins (MMAE, MMAF, AF-HPA) Maytansinoids (DM1, DM4)	Brentuximab vedotin, belantamab mafodotin, trastuzumab emtansine, mirvetuximab soravtansine-gyxn
DNA-damaging payloads	DNA cross-linkers: form covalent cross-links in double-stranded DNA, preventing strand separation and normal replication Topoisomerase inhibitor payloads: inhibit normal function of topoisomerase I in DNA unwinding/replication, inducing apoptosis DNA cleaving agents: bind to DNA’s minor groove and cut the double helix	Calicheamicin: moderate PBD: strong Duocarmycin: moderate	Calicheamicin Duocarmycin PBD dimers SN-38 Dxd Exatecan	Loncastuximab tesirine Sacituzumab govitecan Trastuzumab deruxtecan
Immunomodulatory molecules	Activate innate and adaptive immune pathways (e.g., TLR, STING) or modulate immune cell function	No	LR agonists (TLR7/8) STING agonists GCs and derivatives	ABBV-3373 (not yet approved)

ADC: Antibody-drug conjugate; AF-HPA: auristatin F-hydroxypropylamide; DM1: derivative of maytansine 1; DM4: derivative of maytansine 4; Dxd: deruxtecan; GCs: glucocorticoids; MMAE: monomethyl auristatin E; MMAF: monomethyl auristatin F; PBD: pyrrolobenzodiazepine; SN-38: 7-ethyl-10-hydroxycamptothecin; STING: stimulator of interferon genes; TLR: toll-like receptor; TLR7/8: toll-like receptor 7/8.

## THERAPEUTIC APPLICATIONS AND LIMITATIONS OF ADCS IN CANCER AND AUTOIMMUNE DISEASES

### ADC therapy for malignant tumors

As a novel precision therapeutic strategy, ADCs have demonstrated significant potential for application in various types of malignant tumors in recent years. By targeting tumor-associated antigens and delivering highly potent cytotoxic payloads, ADCs can achieve selective killing of tumor cells while minimizing toxic damage to normal tissues^[22,32,33,50]^.

In hematological malignancies, ADCs precisely recognize and bind to differentiation antigens highly expressed on the surface of malignant cells, thereby achieving high-intensity killing at lower doses^[3,51,52]^.

In acute myeloid leukemia (AML), the target CD33 has garnered widespread attention. In fact, CD33 is not relevant only for hematologic malignancies. Typical ADC drugs such as Gemtuzumab Ozogamicin utilize an anti-CD33 monoclonal antibody to precisely deliver the DNA-damaging molecule calicheamicin into tumor cells. Following internalization, the payload is released in the acidic environment of lysosomes, thereby inducing double-strand DNA breaks and triggering apoptosis^[53-55]^.

For ALL, the primary target is the CD22 molecule. Inotuzumab ozogamicin (INO) is an ADC targeting CD22. After internalization into the lysosomes of tumor cells, calicheamicin is released and binds to DNA, leading to apoptosis. A Stage III clinical study (NCT01564784) has shown that INO exhibits an overall response rate (ORR) of 80.7% in patients with relapsed or refractory ALL, with 78.4% of these patients achieving minimal residual disease (MRD) negativity^[56,57]^.

For lymphoma, molecules such as CD19, CD22, CD30, and CD79b are commonly targeted, highly expressed molecules. Loncastuximab tesirine has demonstrated significant single-agent antitumor activity in patients with relapsed/refractory diffuse large B-cell lymphoma (DLBCL), with an ORR of 48.3% and a complete response (CR) rate of 24.1%. The Polatuzumab vedotin in combination with bendamustine and rituximab (pola-BR) regimen achieved a CR rate of 40.0%, significantly higher than the 17.5% in the control group, and significantly prolonged patients’ progression-free survival (PFS) and overall survival (OS)^[33,51,58-62]^.

Compared to the relatively homogeneous target expression in hematological malignancies, solid tumor therapy faces challenges such as tumor heterogeneity, drug penetration, and uneven target expression. However, ADCs continue to show broad therapeutic promise in multiple cancer types^[22,63]^.

In breast cancer, ADC drugs have become a breakthrough in targeted therapy. trastuzumab emtansine (T-DM1) was the first approved HER2-targeting ADC, which precisely identifies HER2-positive tumor cells via its antibody component and delivers emtansine intracellularly. Trastuzumab deruxtecan (T-DXd), through improved linker design and a more potent topoisomerase I inhibitor, has demonstrated broader applicability, especially in patients with HER2-low breast cancer, by killing adjacent HER2-negative cells via the “bystander effect”. Sacituzumab govitecan has been approved for treating patients with refractory or metastatic triple-negative breast cancer (TNBC)^[64-68]^.

In lung cancer, ADC drugs have demonstrated significant therapeutic potential against various targets. In non-small cell lung cancer (NSCLC), HER2 and Trop-2 are important targets. T-DXd, as a HER2-targeting ADC, has shown notable antitumor activity. Sacituzumab govitecan, by targeting Trop-2, is particularly suitable for patients with relapsed or refractory disease. In small cell lung cancer (SCLC), approximately 80% of patients have high DLL3 expression, and Rovalpituzumab tesirine has demonstrated an ORR of 38% in patients with high DLL3 expression^[69-72]^.

In urothelial carcinoma, enfortumab vedotin is an ADC drug targeting Nectin-4, which has been approved for the treatment of refractory metastatic bladder cancer. Clinical trials have shown that enfortumab vedotin exhibits high response rates and significant survival benefits in bladder cancer patients who are platinum-resistant or have failed immunotherapy.

Overall, ADCs in cancer treatment, through the combination of precise targeting and potent payloads, not only extend patient survival time but also provide more options for patients with different molecular subtypes of tumors, serving as an important bridge connecting targeted therapy and chemotherapy^[73,74]^.

However, as ADCs are widely applied across various oncological indications, a series of structural limitations and mechanistic challenges have gradually been exposed in clinical practice. Especially when expanding to non-malignant indications such as autoimmune diseases, how to maintain the balance between efficacy and safety is becoming a central focus of research.

## ADC THERAPY FOR AUTOIMMUNE DISEASES

In recent years, the exploration of ADC technology in the field of autoimmune disease treatment has been gaining momentum, offering new therapeutic strategies for these diseases, which are often characterized by a long course, complex mechanisms, and significant side effects associated with existing therapies. The core issue in autoimmune diseases is the immune system erroneously attacking self-tissues or maintaining an abnormal inflammatory state. The precision delivery characteristic of ADCs enables the targeted delivery of immunomodulatory agents to specific pathogenic cells or inflammatory sites, achieving effective intervention while significantly reducing systemic side effects^[2,6]^.

The treatment of RA is the most mature area for ADC application in autoimmune diseases. ABBV-3373, as the first anti-inflammatory ADC to enter clinical trials, couples a novel glucocorticoid receptor modulator (GRM) with an anti-TNF monoclonal antibody via an MP-Ala-Ala linker, achieving targeted delivery of glucocorticoids^[75]^. In mouse models of collagen-induced arthritis, this ADC not only significantly suppressed inflammatory responses but also effectively alleviated joint swelling and tissue destruction^[76,77]^. More importantly, a Phase II clinical trial (NCT03823391) showed that, compared to adalimumab, ABBV-3373 demonstrated a greater advantage in improving disease activity scores (-2.65 *vs.* -2.13, *P* = 0.022) and had a lower incidence of adverse events (35% *vs.* 71%), providing a new paradigm for the precision application of glucocorticoids.

In addition to TNF-targeting strategies, CD30-targeting BV has also shown potential in RA treatment. Based on the characteristic significant elevation of soluble CD30 levels in the serum and synovial fluid of RA patients, BV induces apoptosis in CD30-positive cells via its MMAE payload, exhibiting dose-dependent anti-inflammatory effects in mouse arthritis models. Clinical case reports further confirm that BV can achieve dual remission when treating patients with RA complicated by Hodgkin’s lymphoma, suggesting its multiple mechanisms of action in RA treatment^[78-80]^. Furthermore, an A7R-ADC targeting IL-7R, a CD64-calicheamicin conjugate targeting FcγRI, and a siRNA conjugate strategy targeting complement C5 have all shown significant anti-inflammatory effects in animal models, providing a basis for multi-target therapeutic strategies in RA^[81]^.

In the treatment of SLE, ADC research focuses on inhibiting the aberrant activation of plasmacytoid dendritic cells (pDCs). Based on the critical role of pDCs in aberrantly secreting type I interferons and pro-inflammatory cytokines in SLE pathogenesis, researchers have developed BDCA2-ADC (DB-2304), which utilizes a novel Immune Modulating Antibody Conjugate (DIMAC) platform to couple a glucocorticoid with an anti-BDCA2 antibody, achieving highly selective delivery to pDCs. *In vitro* experiments showed that this ADC almost completely blocked IFN-α production in pDCs during TLR stimulation and broadly suppressed pro-inflammatory cytokine expression by agonizing glucocorticoid receptor transcription, holding promise as the first ADC drug for SLE treatment^[82-84]^. Another clinical case report also showed that BV combined with Nivolumab could achieve complete remission in treating SLE complicated by Hodgkin’s lymphoma, further supporting the potential value of BV in SLE treatment, although this notion still lacks support from larger sample sizes and mechanistic studies^[85]^.

In the treatment of systemic sclerosis (SSc), based on the pathological features of elevated soluble CD30 levels in the serum of SSc patients and Th2-like cell activation, BV has emerged as a promising candidate drug for treating this disease. A Phase IIb clinical trial (NCT03222492) showed that BV treatment in patients with severe, active diffuse cutaneous SSc achieved the primary endpoint (a decrease of ≥ 8 in the modified Rodnan skin score) within 24 weeks, demonstrating the efficacy of CD30-targeting strategies in treating fibrotic diseases and suggesting the promise of BV in SSc treatment^[86,87]^.

The exploration of ADCs for other autoimmune diseases is also continuously expanding. Researchers have developed various ADC strategies targeting specific immune cells: an antibody-dexamethasone conjugate targeting CD163 specifically targets macrophages at inflammatory sites, significantly reducing TNF-α secretion without systemic side effects^[88]^; an antibody-dexamethasone conjugate targeting E-selectin targets activated endothelial cells, effectively downregulating pro-inflammatory factor expression^[89]^; an antibody-PDE4 inhibitor conjugate targeting CD11a selectively targets immune cells, showing good anti-inflammatory activity in inflammation models^[90]^. Furthermore, besides ABBV-3373, a strategy coupling a novel glucocorticoid receptor agonist with an anti-CD74 antibody via a pyrophosphate acetal linker, and a glucocorticoid conjugate targeting CD70, have both demonstrated the feasibility of targeted glucocorticoid delivery in *in vitro* experiments^[91,92]^.

### Major challenges and limitations of ADCs in therapy

Currently, despite the aforementioned advantages of ADCs, this therapeutic modality still faces considerable limitations, such as complex pharmacokinetics, numerous adverse reactions, and manufacturing and translational hurdles^[37,93,94]^. Furthermore, when applied to the treatment of autoimmune diseases, ADCs also encounter a considerable number of additional challenges. Beyond the traditional adverse reactions and delivery obstacles, drug resistance, as a more profound limiting mechanism, is also becoming increasingly prominent in ADC therapy. Its mechanisms are complex and heterogeneous, and it has become a core obstacle to limited clinical efficacy. This will be systematically elaborated on in Section 5.

#### Pharmacokinetic properties

The pharmacokinetics of ADCs are highly complex because ADCs circulate in three main forms: the intact ADC, the unconjugated antibody, and the free payload. This unique conjugated structure profoundly alters the half-life, metabolism and clearance, as well as the biodistribution and composition of each component^[95]^. Once decomposition occurs, the antibody portion is broken down into amino acids, while the payload is cleared predominantly via renal and/or hepatic pathways. As a result, patients with impaired liver or kidney function may metabolize ADCs differently, which in turn affects the half-life. While unconjugated monoclonal antibodies generally have half-lives of 1 to 3 weeks, ADCs often exhibit significantly shorter half-lives of just only 2 to 5 days. From this standpoint, constructing pharmacokinetic models to describe the clinical behavior of ADCs - and using these models to guide ADC design - presents a significant challenge^[96]^. Recent reviews have comprehensively summarized these challenges and modeling strategies, offering valuable frameworks for understanding ADC pharmacokinetics in clinical development^[97]^.

#### Side effects

Common adverse events observed with clinically used ADCs include systemic symptoms, neurotoxicity, hematological toxicity, cardiovascular toxicity, hepatotoxicity, ocular toxicity, and hypersensitivity reactions. Among these, severe adverse events (Grade ≥ 3) typically involve systemic symptoms, neurotoxicity, hematological toxicity, hepatotoxicity, and cardiovascular toxicity. Neurotoxicity and cardiovascular toxicity require particularly close attention; the former can significantly impair patients’ quality of life, whereas the latter can threaten life itself. Although ocular toxicity is generally not life-threatening, it can still substantially affect patients’ daily activities and adherence to treatment. For hepatotoxicity and hematological toxicity, careful monitoring is essential to avoid cumulative damage or treatment interruption^[25,45,98-101]^.

In clinical practice, physicians must consider patient-specific factors, potential risks, and the nature of the underlying disease. During treatment, enhanced monitoring and timely intervention are crucial, and patients should be fully informed about possible adverse events. This approach can help optimize safety and improve treatment adherence. Table 4 lists common severe adverse events and their associated ADCs.

**Table 4:** Common severe adverse events and their associated ADCs

**Adverse event type**	**Severity**	**ADCs associated**	**OR (95%CI)**
Systemic	Medium-high	Broad	Fatigue: 1.25 (1.08-1.45) Anorexia: 1.36 (1.09-1.69)
Neurological	High	Brentuximab vedotin, polatuzumab vedotin	Sensory neuropathy 2.18 (1.27-3.76)
Cardiovascular	Medium-high	Heart failure: brentuximab vedotin Cardiomyopathy: trastuzumab emtansine, trastuzumab deruxtecan Embolism: gemtuzumab ozogamicin, inotuzumab ozogamicin, enfortumab vedotin Ventricular arrhythmia: polatuzumab vedotin, sacituzumab govitecan	Heart failure: 1.748 (1.351-2.262) Cardiomyopathy: 4.766 (2.759-8.235), 4.433 (3.144-6.251) Embolism: 11.806 (10.259-13.587), 29.67 (24.242-36.314), 1.628 (1.023-2.591) Ventricular arrhythmia: 2.302 (0.862-6.144), 3.013 (0.969-9.367)
Hematological	High	Gemtuzumab ozogamicin, inotuzumab ozogamicin, trastuzumab emtansine	Thrombocytopenia: 2.07 (1.00-4.31), Neutropenia: 0.56 (0.31-1.01)
Hepatic	High	Trastuzumab emtansine, sacituzumab govitecan	Increased ALT: 2.51 (1.84-3.40), Increased AST: 2.83 (2.04-3.93)

ADC: Antibody-drug conjugate; OR: odds ratio; CI: confidence interval; ALT: alanine aminotransferase; AST: aspartate aminotransferase.

#### Unique challenges in the application of ADCs in autoimmune diseases

Although ADCs have made significant progress in the field of cancer therapy, translating this technology platform to the realm of autoimmune diseases presents a series of unique challenges. A fundamental distinction from oncology is the treatment paradigm: autoimmune diseases typically require chronic, often lifelong therapy and patients are often not facing an immediate life-threatening urgency. This imposes higher demands on the safety and selectivity of ADCs^[102]^.

Special Considerations for Target Selection: In autoimmune diseases, selecting an appropriate target is the primary challenge for ADC development. Unlike in the oncology field, there is a lack of targets in autoimmune diseases that are truly parallel to “tumor-specific antigens”, making “off-target effects” a more prominent issue. Therefore, an ideal ADC target for autoimmune diseases should meet three key criteria: (1) high enrichment on pathogenic immune cells; (2) limited expression on normal immune cells; and (3) efficient internalization to deliver the drug^[103]^. Ideally, ADCs need to target autoreactive immune cell subpopulations rather than the entire population of that subset, but reliable surface markers to distinguish these pathogenic and protective subpopulations are currently lacking^[104]^.

Safety Considerations for Chronic Treatment: The chronic nature of autoimmune diseases dictates that ADC therapy may require long-term or intermittent administration, which is in stark contrast to the finite treatment courses in oncology. This fundamental difference raises several key safety concerns: (1) Risk of cumulative toxicity: Even with low doses of cytotoxic drugs, long-term exposure can lead to cumulative toxicity. Research by Nguyen *et al*. indicates that even minute payload leakage can accumulate during long-term treatment, leading to myelosuppression, neurotoxicity, or hepatotoxicity^[105]^. This necessitates more cautious payload selection for autoimmune ADCs, potentially prioritizing non-cytotoxic but immunomodulatory payloads and more stable linkers; (2) Balancing immune system suppression: Autoimmune ADCs must strike a balance between suppressing pathogenic immune responses and maintaining normal immune function. Excessive suppression can increase the risk of infections and malignancies, especially in the context of long-term therapy. This challenge is relatively minor in oncology ADC therapy, as short-term immunosuppression is generally acceptable for patients^[105]^.

The clinical experience with ABBV-3373 provides valuable insights. This ADC combines an anti-TNF antibody with a GRM, aiming for more precise immunomodulation. Studies by Buttgereit *et al*. have shown good short-term safety and efficacy, but its long-term safety and immunogenicity still require more data support^[106]^. Research by D’Cunha *et al*. further revealed ABBV-3373’s favorable pharmacokinetic properties, manageable immunogenicity, and overall high tolerability, providing a basis for its application in autoimmune diseases^[75]^.

Although ABBV-3373 shows good promise, its payload choice (a GRM) essentially falls within the category of traditional anti-inflammatory drugs. This reflects a conservative trend in current autoimmune ADC development - a preference for using known immunomodulators rather than exploring entirely new mechanisms of action. While this strategy reduces development risks, it may also miss opportunities to discover revolutionary therapeutic methods. Whether novel payloads, such as epigenetic regulators or cell metabolism modulators, should be explored more boldly in the future is worthy of deep consideration by the industry.

In conclusion, the application of ADCs in autoimmune diseases represents a challenging yet promising frontier. Overcoming challenges in target selection, long-term safety, and translational hurdles will be key to realizing this potential. As hinted by Dixit *et al*., the success of autoimmune ADCs will not merely be a replication of the successes in oncology but will require innovation tailored to the unique biology and clinical needs of autoimmune diseases^[107]^. It needs to be recognized that current efforts to translate ADCs from oncology to autoimmune diseases may suffer from fundamental conceptual limitations. ADCs were initially designed to “kill” target cells, whereas autoimmune diseases more often require “modulation” rather than “elimination”. Simply replacing cytotoxic payloads with immunomodulators may not fully exploit the potential of the ADC platform. Perhaps a fundamental rethinking of the mode of action for ADCs in autoimmune diseases is needed - for instance, developing ADCs based on entirely new concepts such as reversible modulation, spatiotemporally specific release, or cell reprogramming, thereby unleashing the novel potential of the ADC platform in the field of immunomodulation.

## ADC RESISTANCE MECHANISMS

ADCs, which combine the targeting specificity of monoclonal antibodies with the cytotoxicity of small-molecule toxins, have made remarkable progress in the treatment of diseases such as cancer and autoimmune diseases. However, the emergence of ADC resistance in clinical practice has become a significant challenge limiting their therapeutic efficacy. Whether through intrinsic resistance or acquired resistance following treatment, target cells can employ multiple distinct mechanisms to evade the cytotoxic effects of ADCs, leading to cancer treatment failure. Therefore, a thorough elucidation of ADC resistance mechanisms and the exploration of strategies to overcome this resistance are of significant clinical importance for enhancing ADC efficacy and prolonging patient survival^[108,109]^.

### Antigen-related resistance

Sufficient target antigen expression on tumor cells is a prerequisite for ADC efficacy. Reduction in the quantity and alterations in the distribution of the target antigen are the most direct factors contributing to ADC resistance. Studies have found that after long-term exposure to T-DM1, HER2 protein levels significantly decrease in resistant cell lines, suggesting that continuous ADC pressure can lead to target antigen downregulation^[110,111]^.

Spatial heterogeneity of the target antigen within tumors can also impair ADC efficacy. Spatial heterogeneity in tumor cells leads to significant differences in antigen expression across different tumor regions. Even with an overall determination of “HER2 high expression”, a substantial number of HER2-negative subpopulations may still exist, reducing ADC penetration depth and coverage. In the ZEPHIR study, PET imaging using radiolabeled antibodies to assess HER2 distribution revealed that in approximately 46% of patients, less than half of the tumor regions showed uptake of the tracer antibody^[112]^. In such cases, the ADC and its bystander effect may be insufficient to completely eradicate antigen-negative target cells. As treatment progresses, target cells may develop new mutations under selective pressure, thereby affecting antigen expression and cell tolerance^[66-68,113]^.

Changes in the nature of the target antigen can also mediate resistance. “Antigen drift”, where the target antigen undergoes structural changes due to gene mutations, leading to difficulties in ADC recognition, is one such instance. In anti-HER2 therapy, tumor cells can express a truncated form of HER2 (p95HER2) that lacks the extracellular domain, reducing the efficacy of trastuzumab-based drugs^[114]^. After treatment with the anti-CD22 ADC INO in ALL, 11% of relapsed patients developed acquired CD22 mutations, including forms such as protein truncation and epitope alterations^[115]^.

### Impaired drug transport and efflux mechanisms

For an ADC to be effective, it must be internalized by tumor cells and release its payload intracellularly. The ADC cannot function if it is not efficiently internalized, if the payload is not effectively released, or if it is expelled from the cell before it can act.

Alterations in endocytic pathways are a significant resistance mechanism. Under normal conditions, most ADCs enter lysosomes via clathrin-mediated endocytosis. However, resistant cells can alter this pathway, leading to resistance^[108,110,116]^. For instance, *in vitro* studies by Sung *et al*. found that T-DM1-resistant cells overexpress Caveolin-1 (CAV-1), causing HER2 receptors to be internalized primarily through the caveolae-mediated pathway. This results in reduced co-localization of the antibody-drug complex with lysosomes, thereby diminishing the cytotoxic effect of T-DM1^[117]^.

Lysosomal dysfunction itself can also affect the release of the cytotoxic payload. Decreased activity of proteolytic enzymes or insufficient acidification within lysosomes can lead to incomplete antibody degradation, preventing the full release of the payload. Ríos-Luci *et al*. reported that some T-DM1-resistant cells exhibit elevated lysosomal pH, leading to impaired processing of T-DM1 within the lysosome. This highlights the role of maintaining lysosomal acidity in combating resistance^[118]^.

For ADCs with non-cleavable linkers, payload release is more dependent on specific transport mechanisms. The specific metabolite Lysine-MCC-DM1, generated after the lysosomal degradation of T-DM1, requires the lysosomal membrane protein SLC46A3 for its export. Research by Tomabechi *et al*. found that effective SLC46A3 inhibitors, such as clarithromycin, erythromycin, and rifabutin, can significantly reduce T-DM1 cytotoxicity. This is consistent with the fact that downregulation of SLC46A3 expression in cancer cells is associated with T-DM1 resistance. Low SLC46A3 expression is correlated with the T-DM1 resistance phenotype and can serve as a predictive biomarker for its efficacy^[119]^.

Tumor cells can significantly reduce the cytoplasmic concentration of payloads by upregulating drug efflux pumps. ATP-binding cassette (ABC) transporters (e.g., P-glycoprotein/MDR1, BCRP/ABCG2) actively expel hydrophobic chemotherapeutic drugs from the cell, thereby reducing the intracellular concentration of the active drug. Commonly used payloads (including vinca alkaloid/taxane-type tubulin polymerization inhibitors and camptothecin-type topoisomerase inhibitors) are known substrates of ABC transporters^[41,120,121]^.

P-gp (MDR1) is one of the most extensively studied ABC transporters, and it can recognize and pump out auristatin- and maytansinoid-class payloads. The DM1 payload of T-DM1 can be effluxed by P-gp, and resistant cells often exhibit P-gp upregulation. Li and Wang *et al*. also found that resistant breast cancer clones showed cross-resistance to free DM1 and vinca alkaloids, accompanied by upregulation of the *MDR1* gene, indicating the role of P-gp-mediated efflux^[122,123]^.

Overall, ABC transporter-mediated drug efflux is one of the common mechanisms of ADC resistance. When this mechanism is present, simply increasing the ADC dosage is often of little benefit, as the cells can rapidly pump out the toxin. To address this situation, researchers are exploring strategies such as using drugs that are less easily recognized by efflux pumps or co-administering efflux inhibitors to overcome MDR-mediated ADC resistance (see Section 6 for details).

### Mechanisms of cell death pathway inhibition

Even if ADCs successfully deliver their toxic payload into the cell, tumor cells can still evade apoptosis by modulating damage response and cell death pathways. Dysfunction of apoptotic signaling pathways is a common mechanism of tumor resistance to various therapies, and ADC resistance is no exception. In cases of resistance to INO in acute leukemia, inactivating mutations in tumor suppressor genes such as TP53 and ATM are often detected^[115]^. Furthermore, tumor cells can counteract the effects of the toxin by upregulating survival signals or anti-apoptotic proteins. For example, overexpression of anti-apoptotic proteins such as BCL-2 and BCL-XL can render tumor cells insensitive to apoptotic stimuli mediated by Gemtuzumab Ozogamicin or BV. In preclinical models, combining these ADCs with BCL-2 inhibitors can partially restore their killing effect, supporting the role of anti-apoptotic pathways in resistance^[124,125]^.

On the other hand, resistance and adaptation targeting the site of action of the ADC’s payload can also lead to resistance. For example, the DM1 payload of T-DM1 targets microtubules, and long-term administration may lead tumor cells to develop resistance by modulating microtubule-associated molecules. This includes mutations in β-tubulin genes, alterations in isotype expression profiles, or upregulation of microtubule-stabilizing proteins, thereby attenuating DM1’s interference with microtubule dynamics. For topoisomerase I inhibitor payloads, tumor cells can counteract toxicity by enhancing their DNA damage repair capabilities. For instance, with DXd, the active metabolite of DS-8201, studies have found loss-of-function mutations in the DNA repair-related gene SLX4 (an assembly scaffold protein for endonucleases) in tumors. These mutations cause cells to respond differently to DNA damage induced by topoisomerase inhibition, thereby avoiding cell death^[126]^.

Thus, when cellular signal transduction and damage response to the toxin are altered, the lethal effect of ADCs is greatly diminished. These mechanisms enable resistant cells to bypass the execution of apoptotic programs in response to ADC-induced damage, thereby evading death. In the future, combining ADCs with pro-apoptotic drugs or drugs targeting repair pathways may potentially restore the sensitivity of such resistant cells to ADCs.

### Specificity of ADC resistance mechanisms in autoimmune diseases

Although ADCs face resistance challenges in both autoimmune diseases and cancer treatment, the pathological characteristics and therapeutic needs of autoimmune diseases dictate that their resistance mechanisms possess significant uniqueness beyond the traditional ADC resistance observed in oncology.

The immunogenicity of ADCs may be a prominent and unique challenge in their therapeutic application. Research by Pizano-Martinez *et al*. shows that the incidence of anti-drug antibodies (ADAs) induced by biologics in patients with autoimmune diseases can be as high as 40%^[127]^. This difference stems from the pre-existing overactivated state of the immune system in autoimmune disease patients, making them more prone to generating immune responses against exogenous proteins. Carrasco-Triguero noted that while ADA generation does not necessarily imply a reduction in ADC titer or therapeutic efficacy, there are indeed cases where the corresponding ADC is undetectable in patients with elevated ADAs^[128]^.

Cross-reactivity is another immunogenicity challenge. Carrasco-Triguero’s research indicates that although patients generally develop ADAs only against the mAb, they might also simultaneously produce antibodies against the antibody, linker, and payload, increasing the risk of immunogenicity. In autoimmune diseases, such multi-component immune responses may be more complex, as the patient’s immune system has already mounted abnormal responses to multiple self-antigens and might more readily recognize and attack different components of the ADC^[128]^.

Another difficulty is the highly heterogeneous and dynamically changing nature of pathogenic immune cell populations in autoimmune diseases. With the advancement of single-cell transcriptomic technologies, an increasing number of studies have noted the heterogeneity of pathogenic immune cell expression in autoimmune diseases. As Zhao *et al*. pointed out, these newly discovered cell subtypes may suggest new therapeutic targets^[129]^. However, this also places higher demands on the design of novel ADCs; if these rare cell subpopulations cannot be targeted, the desired therapeutic outcomes may not be achieved, thereby affecting the ADC’s therapeutic coverage and durability of treatment.

In summary, ADC resistance mechanisms in autoimmune diseases exhibit significant uniqueness, primarily manifested in increased immunogenicity risks and dynamic changes in immune cell subpopulations. These unique mechanisms necessitate the adoption of strategies different from those for oncology ADCs when developing ADCs for autoimmune diseases, including more stable molecular designs, lower immunogenicity, multi-target coverage, and personalized treatment regimens. Only by fully recognizing and addressing these unique challenges can ADCs exert their potential advantages in the treatment of autoimmune diseases.

## STRATEGIES TO OVERCOME ADC RESISTANCE AND FUTURE DIRECTIONS

The challenge of resistance in ADC therapy has spurred researchers to continuously innovate in both molecular design and therapeutic strategies, exploring methods to overcome resistance and future developmental directions. This section will focus on three main themes: molecular-level strategy innovations, therapeutic strategy innovations, and the challenges of resistance and countermeasures in the application of ADCs for autoimmune diseases.

### Molecular-level strategy innovations

Innovation at the molecular design level is the most direct and effective approach to overcoming ADC resistance. Through meticulous refinement of the three core components of ADCs - the antibody, linker, and payload - the drug’s anti-resistance capabilities can be fundamentally enhanced. This subsection will primarily discuss how to bypass transporter-mediated resistance mechanisms through optimized payload and linker design, as well as how to utilize multi-target and dual-payload strategies to broaden the killing spectrum and reduce the risk of resistance.

The initial focus is on the optimization of ADC components, namely the antibody, linker, and payload. The choice of antibody directly influences the targeting specificity and tumor penetration of an ADC. In recent years, small-format antibody fragments [such as Fab fragments, single chain variable fragments (scFv), and designed ankyrin repeat proteins (DARPins)] have demonstrated unique advantages in novel ADC designs^[22,130,131]^. Furthermore, modifications to the Fc region can also alter functions such as the ADCC activity of ADCs. For example, the S239D/I332E mutations in the Fc region have been demonstrated to significantly improve binding to FcγRIIIa, thereby enhancing ADCC^[132]^.

Modern linker design thus may emphasize not only stability in the bloodstream but also specific drug release in the target organ microenvironment. As mentioned earlier, cleavable linkers can take advantage of differences between the intracellular and extracellular environment, ensuring that payload release mainly occurs within target cells. While linkers responsive to a single stimulus (e.g., pH or a specific protease) have already shown clear benefits in ADC design, multifunctional “intellective” linkers that combine multiple stimuli offer even higher precision in drug release. Some linkers integrate acid responsiveness with protease sensitivity, enabling them to be “partially activated” under low-pH conditions and subsequently cleaved by tumor-associated enzymes. For example, hydrazone bonds respond to acidic pH, making them active in the mildly acidic tumor environment^[133]^. Meanwhile, Cathepsin B, which is often overexpressed in tumor cells, specifically recognizes and cleaves linkers containing enzyme-sensitive sequences (such as Val-Cit). Such linkers enable a two-step release mechanism: partial activation in the acidic microenvironment, followed by complete payload release upon Cathepsin B cleavage^[133-135]^.

Additionally, the development of site-specific conjugation technologies for linkers has made it possible for each antibody molecule to carry a uniform number of payloads (resulting in a homogeneous drug-to-antibody ratio, DAR), enabling positional control, maximally reducing product heterogeneity, and preserving key epitope-binding regions^[94]^. Of course, the limitations of such strategies lie in higher demands on antibody engineering and conjugation chemistry, and correspondingly increased production costs. These innovations are expected to further enhance the efficacy of ADCs against resistant tumors while maintaining safety, leading to more refined ADC molecular designs.

As previously mentioned, resistance to many ADCs stems from the overexpression of ABC transporters (such as P-gp) by tumor cells. To address this mechanism, Kovtun *et al*. designed an ADC in which the traditional hydrophobic SMCC linker was replaced with a hydrophilic PEG4-Mal linker. While carrying the same DM1 toxin, this hydrophilic linker-ADC exhibited higher cytotoxic activity in MDR1-overexpressing tumor models, successfully bypassing P-gp-mediated resistance^[41]^. In fact, such masking groups can not only enhance efficacy but also reduce non-specific interactions of ADCs with normal tissues, mitigating side effects such as aggregate formation, rapid clearance, and potential high immunogenicity caused by highly hydrophobic payloads.

To enhance the killing spectrum, another key strategy involves engineering bispecific and dual-payload ADCs. These next-generation designs represent a critical direction for addressing resistance driven by antigen loss or signaling pathway compensation. Although these new types of ADCs are currently in early clinical or preclinical research stages, the results presented so far have been largely encouraging.

Bispecific or multi-specific ADCs, by simultaneously recognizing two antigens, improve tumor binding affinity and internalization efficiency, and can circumvent resistance caused by antigen loss associated with single-target approaches. To date, over 100 different design formats for bispecific ADCs (bsADCs) have been reported in the literature^[136]^. Andreev *et al*. constructed a bispecific ADC that bridges HER2 and the prolactin receptor (PRLR), which can co-target PRLR on the surface of HER2-positive tumor cells, leading to significantly enhanced internalization and killing effects of the ADC in tumors co-expressing HER2 and PRLR^[137]^. Furthermore, combining two non-tumor-specific but complementary targets can also reduce off-target toxicity. For example, Luo *et al*. developed a dual-target ADC for EpCAM and CLDN3, which requires high co-expression of both antigens on tumors for effective killing, exhibiting minimal toxicity to normal cells that only highly express EpCAM but have low CLDN3 levels, thereby alleviating the toxicity limitations of single-target EpCAM ADCs^[138]^. In summary, dual-target ADCs, through a “dual recognition” mechanism, enhance tumor specificity and internalization efficiency, and can, to some extent, overcome resistance caused by antigen heterogeneity or downregulation.

On a parallel track, dual-payload or multi-payload ADCs conjugate two cytotoxic drugs with complementary mechanisms of action onto the same antibody, aiming to achieve synergistic killing and reduce the probability of resistance. Yamazaki *et al*. constructed an anti-HER2 dual-payload ADC carrying both MMAE and MMAF toxins, which achieved complete remission in breast cancer xenograft models with low HER2 expression and resistance to traditional T-DM1, significantly outperforming the efficacy of either single-payload ADC^[139]^. Mechanistically, the more potent MMAF effectively kills resistant cells, while the conjugated MMAE exerts a bystander effect to eliminate nearby antigen-negative tumor cells, thereby enhancing overall efficacy. Kumar *et al*. also synthesized a hetero-trifunctional linker, enabling the simultaneous conjugation of the microtubule inhibitor MMAE and the DNA intercalator PBD dimer onto a single antibody, achieving more efficient killing^[140]^.

Dual-payload ADCs hold the promise of broadening the antitumor spectrum and reducing the chances of tumor cells developing tolerance to a single drug, but their development also faces challenges such as precise control of the DAR and pharmacokinetic balancing. Currently, the world’s first dual-payload ADC to enter clinical trials is KH815 (an anti-TROP2 ADC) developed by China’s Kanghong Pharma (NCT06885645): this drug links two types of toxins, a topoisomerase I inhibitor and an RNA polymerase II inhibitor, to enhance antitumor efficacy by inducing DNA double-strand breaks on one hand and inhibiting RNA synthesis on the other. Interestingly, KH815 was also observed in preclinical studies to downregulate the expression of resistance-related proteins such as P-gp and HSP70 in tumor cells, thereby increasing sensitivity to chemotherapy. Table 5 lists a summary of dual-target and dual-payload ADCs in clinical trials.

**Table 5:** Clinical trial summary of dual-target and dual-payload ADCs

**Drug name (code)**	**Mechanism and target(s)**	**Clinical trial ID**	**Phase**	**Status**	**Published results**
Zanidatamab Zovodotin (ZW49)	Dual-Target ADC (Bispecific HER2)	NCT03821233	I	Recruiting	Preliminary phase 1 showed confirmed ORR across multiple cancer types was 28% and disease control rate was 72%^[141]^
BL-B01D1	Dual-Target ADC (Bispecific EGFR/HER3)	NCT05194982	I	Recruiting	In 174 heavily pretreated patients, ORR was 34%^[142]^
M1231	Dual-Target ADC (Bispecific MUC1/EGFR)	NCT04695847	I	Recruiting	First-in-human trial; no efficacy data have been publicly presented to date
AVZO-1418 (DB-1418)	Dual-Target ADC (Bispecific EGFR/HER3)	N/A	I/II	Not yet recruiting	The trial is expected to initiate in 2025
DB-1419	Dual-Target ADC (Bispecific B7-H3/PD-L1)	NCT06554795	I/II	Recruiting	First-in-human trial initiated in 2024. No clinical results have been publicly presented to date
KH815	Dual-Payload ADC (TROP2-Targeted; Topo-I + RNA Pol II Inhibitor)	NCT06885645	I	Not yet recruiting	First global dual-payload ADC to receive IND clearance. No clinical results have been publicly presented to date
IBI3020	Dual-Payload ADC (CEACAM5-Targeted)	NCT06963281	I	Recruiting	First patient dosed in Q1 2025. No clinical results have been publicly presented to date

ADC: Antibody-drug conjugate; B7-H3: B7 homolog 3; CEACAM5: carcinoembryonic antigen-related cell adhesion molecule 5; EGFR: epidermal growth factor receptor; HER2: human epidermal growth factor receptor 2; HER3: human epidermal growth factor receptor 3; IND: investigational new drug; MUC1: mucin 1; ORR: objective response rate; PD-L1: programmed death-ligand 1; RNA Pol II: RNA polymerase II; Topo-I: topoisomerase I; TROP2: trophoblast cell-surface antigen 2.

It is worth noting that dual-target and dual-payload designs do not necessarily guarantee higher efficacy. Researchers have developed two anti-HER2 ADCs loaded with MMAE + SG3457 and MMAF + PNU-15968, respectively; unfortunately, neither demonstrated superior efficacy in *in vitro* experiments. This suggests that there are more interactions between dual-payload/dual-target ADCs and tumor cells that warrant further exploration, and the generation of their combined therapeutic effects depends on in-depth molecular interaction studies and basic/clinical trials^[140,143]^.

In addition to the classic optimization designs mentioned above, there have also been recent reports on other optimization strategies such as Probody-drug conjugates, Degradative-antibody conjugates, and Immune receptor agonists. However, they are still in very early preclinical research stages and will not be elaborated upon here^[94]^.

### Innovations in therapeutic strategies

The challenge of resistance to ADC monotherapy is prompting a shift in clinical practice toward combination treatment strategies. Synergy between different drugs can not only enhance efficacy but, more importantly, prevent and overcome the development of resistance through multi-mechanistic parallel attacks. This subsection will systematically elaborate on the principles, clinical practice, and prospects of strategies combining ADCs with different types of drugs, such as immune checkpoint inhibitors (ICIs), small molecule targeted drugs, traditional chemotherapy, and emerging cell therapies.

ADCs can be combined with ICIs to exert synergistic antitumor immunity. ADC-mediated tumor cell apoptosis not only directly kills tumor cells but also releases tumor-associated antigens and danger signals, inducing immunogenic cell death (ICD) and activating dendritic cells, thereby enhancing T cell infiltration and tumor-specific immune responses. However, under chronic antigen stimulation, infiltrating T cells often exhibit an exhausted state. Combining ADCs with ICIs can fully leverage the tumor antigen release and immune activation effects triggered by ADCs, with ICIs releasing the “brakes” on T cells for tumor clearance, forming a mechanistic complement between the two^[13,38,144,145]^. T-DXd; DS-8201a, as a novel ADC, has demonstrated significant antitumor activity and potential to overcome resistance when combined with immunotherapy. Currently, multiple clinical trials evaluating the combination of T-DXd with ICIs are underway. Early evidence has shown that the combination of T-DXd and ICIs can elicit antitumor effects exceeding those of chemotherapy or ICI monotherapy in various cancer types. In the BEGONIA Phase Ib/II trial (NCT03742102), durvalumab (a PD-L1 inhibitor) plus T-DXd achieved an ORR of 66.7% in patients with metastatic triple-negative breast cancer (mTNBC), compared to 58.3% in the control group of durvalumab plus paclitaxel, suggesting a quantifiable improvement in efficacy with the addition of T-DXd. In the urothelial cancer cohort of the DS8201-A-U105 Phase Ib study (NCT0352357), nivolumab (a PD-1 inhibitor) plus T-DXd achieved an ORR of 36.7%, including 4 CR and 7 partial responses, with a median duration of response of 13.1 months, demonstrating a depth and durability of response difficult to achieve with traditional regimens. Mechanistically, T-DXd enhances tumor antigen presentation and CD8^+^ T cell infiltration through ADCC and the “bystander effect”, providing an ample effector cell basis for ICIs to lift immune suppression, thereby creating synergistic amplification. These phenomena reveal that ADCs can “turn” “cold tumors”, which are traditionally insensitive to immunotherapy, into “hot tumors” rich in T cell infiltration, thereby enabling patients who have partial responses or resistance to ADC monotherapy to achieve more comprehensive and durable tumor control^[146]^.

While this combination therapy has not yet been shown to produce novel, uncontrollable toxicities, the potential for overlapping toxicities remains a key consideration, as both ADCs and ICIs can independently cause immune-related adverse events (irAEs)^[147]^. Optimizing the administration sequence and dosage, and identifying the most synergistic yet safely manageable combinations, will be key to the successful application of this strategy in the future.

Combining ADCs with small-molecule targeted drugs or chemotherapy offers another promising strategy to overcome resistance. Because resistant tumors often exhibit complex mechanisms such as poly-clonality and signaling pathway compensation, which are difficult to control comprehensively with a single agent. Combining ADCs with other antitumor drugs can leverage differences in their mechanisms of action to achieve better synergistic effects, a strategy widely considered effective for overcoming resistance^[148]^.

In terms of molecular targeting, for ADC-resistant cells exhibiting cell cycle dysregulation or signaling abnormalities, combining ADCs with small molecule drugs that inhibit the corresponding pathways can restore ADC sensitivity. In HER2-positive breast cancer, Cyclin E overexpression can lead to T-DM1 resistance^[149]^. Preclinical studies by Witkiewicz *et al*. found that the addition of CDK4/6 inhibitors can arrest excessive tumor cell proliferation and restore T-DM1-induced mitotic arrest and killing effects^[150]^. Similarly, as previously mentioned, overexpression of anti-apoptotic proteins like BCL-2 and BCL-XL can render cells insensitive to ADCs. *In vivo* studies by Zoeller *et al*. further confirmed that neutralizing BCL-2/BCL-xL can enhance T-DM1’s killing of resistant tumors and prolong survival. This indicates that for resistant cells reliant on anti-apoptotic pathways or cell cycle escape, combining ADCs with corresponding small molecule inhibitors (such as BCL-2 inhibitors, PLK1 inhibitors, *etc*.) is a feasible approach^[151]^.

Regarding chemotherapy, combining ADCs with traditional chemotherapeutic drugs also shows complementary advantages: The Phase II TEAL study demonstrated that T-DM1 combined with lapatinib and albumin-bound paclitaxel significantly increased the pathological CR rate in patients, exhibiting a stronger tumor-shrinking effect compared to traditional “dual antibody + chemotherapy” regimens^[152]^. For instance, early studies (BP22572/NCT00934856) found that combining T-DM1 with the classic taxane drug docetaxel could also enhance efficacy, but with more severe adverse reactions. This suggests that in designing ADC + traditional chemotherapy combination regimens, careful consideration must be given to balancing dosage and treatment duration to avoid severe adverse reactions. Overall, rational drug pairing and optimization of administration sequence based on resistance mechanisms are expected to maximize the efficacy of combination therapies and reduce the incidence of resistance^[153]^.

Furthermore, the integration of ADCs with other emerging therapies is also an area of exploration. For example, linking ADC therapy with adoptive cell immunotherapies such as CAR-T cells is thought to potentially overcome extreme cases of resistance. CAR-T cell therapy has achieved breakthrough success in hematological malignancies but is often limited in solid tumors due to antigen heterogeneity and an immunosuppressive microenvironment. When tumor cells “escape” ADC attack due to antigen negativity, CAR-T cells targeting different antigens can be considered to eliminate remaining cancer cells, achieving complementarity.

Kim *et al*. further validated the feasibility of this synergistic strategy by establishing CEACAM5-expressing NSCLC models. When the anti-CEACAM5 ADC SAR408701 showed limited efficacy or resistance, CAR-T cells targeting the same antigen could still effectively induce cytotoxic responses and significantly inhibit tumor growth *in vivo*^[154]^. This indicates that CAR-T therapy can serve not only as a supplementary treatment for ADC-resistant patients but also, in early combination, intervene proactively to reduce the risk of relapse or ADC resistance. Particularly noteworthy is that CAR-T cells have a higher dependency on antigen expression levels, whereas ADCs may, to some extent, rely on the “bystander effect” to kill low-expressing or even antigen-negative tumor cells, thus exhibiting a high degree of mechanistic complementarity. Zhao’s review also proposed from a systemic perspective that this “ADC + CAR-T” dual-track immune attack strategy can not only cover a broader antigen spectrum but also address complex resistance and immune escape mechanisms in the tumor microenvironment. ADCs are responsible for delivering an initial, highly effective cytotoxic strike, while CAR-T cells, relying on immune memory and self-amplification capabilities, achieve sustained clearance, thereby forming a synergistic system of acute strike and long-term immune surveillance^[155]^.

Overall, data on the concurrent or sequential application of ADCs and CAR-T cells are still very limited. Considering the complex preparation and management processes required for CAR-T cell therapy, the feasibility of combining ADCs with it will face more difficulties in clinical research^[156]^. However, it can be speculated that the combined application of multiple therapeutic strategies will be key to overcoming ADC tumor resistance in the future; ADCs, acting as “missiles”, can certainly be combined with immune cells or other drugs to achieve better outcomes. In summary, at the therapeutic strategy level, both the combination with classic drugs and the integration of novel immune/cell therapies have greatly broadened the application boundaries of ADCs, providing a richer arsenal for tackling the problem of resistance. We have reason to expect that after rigorous clinical trial screening, several effective ADC combination regimens will enter the standard of care, helping more patients overcome the challenges posed by drug resistance.

### Unique strategies to address ADC resistance in autoimmune diseases

The application of ADC therapy in autoimmune diseases faces a series of unique challenges distinct from those in oncology, which can lead to reduced actual efficacy or “resistance” phenomena. These include enhanced drug immunogenicity, heterogeneity in target expression at lesion sites, and the safety requirements of long-term medication. Therefore, innovative strategies targeting these specific mechanisms are needed to improve the efficacy and tolerability of ADCs in autoimmune diseases. Several potential key strategies are summarized below.

Reducing the Immunogenicity Risk of ADCs: Patients with autoimmune diseases often require long-term, repeated administration. Even if an ADC formulation has low reported immunogenicity in cancer treatment, the accumulation of minor immune responses during long-term use can lead to the production of ADAs, thereby diminishing efficacy. Consequently, ADC design must aim to minimize immunogenicity. Regarding the requirement to reduce cross-reactivity and immunogenicity risks in autoimmune diseases, current ADC modifications are still focused on the antibody, linker, and payload components. For example, selecting low immunogenicity “small molecule” drugs rather than bacterial/plant toxins, and optimizing linkers to avoid the exposure of new antigenic epitopes, are considered^[103]^. PROTAC payloads, acting as catalytic degraders, can specifically degrade pathogenic proteins at very low doses; their small molecule structure inherently possesses very low immunogenicity, thereby reducing immune system recognition and antibody generation^[157]^. Similarly, antibody-siRNA conjugates deliver RNAi components specifically to lesion cells, silencing pathogenic genes intracellularly and avoiding the off-target damage to non-target cells caused by traditional cytotoxic drugs (chemically modified siRNAs can also inhibit innate immune activation)^[158]^. Furthermore, some studies have attempted to reduce immune system recognition by engineering antibody frameworks to remove T cell epitopes or by using human proteases/enzymes as toxic effectors. Wang *et al*. developed a low-immunogenicity anti-CXCR4 antibody based on a human antibody framework and successfully conjugated the immunosuppressive drug Dasatinib for T cell-specific delivery^[159]^. These low-immunogenicity design strategies are expected to maintain ADC activity during chronic administration while maximally reducing the production of ADAs.

Addressing Heterogeneity in Target Antigen Expression: As previously mentioned, the pathogenesis of autoimmune diseases is complex, involving multiple immune cell subsets and molecular pathways, and the expression of pathogenic targets is highly heterogeneous among different patients or disease stages. Single-target ADCs can hardly cover all pathogenic cell populations, and residual pathogenic cells can lead to disease persistence or relapse. One strategy to address this issue is to refine multi-target combinations and adaptive designs. On one hand, BsADCs can be developed and explored in autoimmune diseases. Although BsADCs are currently studied mainly in oncology, this strategy is equally attractive for combating heterogeneous target cells in autoimmune diseases. On the other hand, personalized multi-target regimens are also an important direction, involving the selection of different ADC combinations or modularly switching targets based on the patient’s pathogenic pathway. In fact, ADCs targeting different immune cells have already shown correspondingly excellent effects^[160]^. Preclinical studies have shown that an anti-E-selectin Dex (Dexa-AbhEsel) ADC, an anti-TNF-α glucocorticoid ADC, and an anti-CD163 Dex (Cymac-001) ADC, used to deliver dexamethasone as an anti-inflammatory payload *in vitro* and *in vivo* respectively, have all achieved positive preclinical results in eliminating dysfunctional immune cells and pro-inflammatory cytokines^[49,88,161]^. The aforementioned development of single-cell transcriptomics and other techniques has identified more rare subpopulations of heterogeneous pathogenic immune cells. While this poses greater challenges for ADC design, it also represents a new direction; new ADC designs may leverage characteristic markers of these rare cell populations obtained from single-cell transcriptomics to achieve broader disease coverage.

Although efforts by the academic community to explore ADC resistance in autoimmune diseases are noticeable overall, we must still recognize that related research remains considerably insufficient. Clinical data capable of supporting evidence-based medicine-level conclusions are extremely scarce, and current explorations face significant difficulties in clinical translation. The keys for the future lie in: establishing unified immunogenicity assessment and follow-up systems; validating safety and efficacy persistence under long-term repeated administration; accurately delineating pathogenic cell lineages using single-cell omics and spatial multi-omics to provide an operational framework for stratified medicine with multi-target or programmable ADCs; and truly translating cutting-edge technologies such as PROTAC-ADCs and siRNA-ADCs into clinical practice and real-world cohorts to form an interdisciplinary closed-loop validation. Only through multi-center, longitudinal, large-sample clinical trials, as well as collaborative iteration between industry and academia, can these conceptual strategies be truly transformed into replicable, affordable, and durably effective therapeutic regimens.

In summary, to address the multidimensional challenges of ADC resistance, scientific research and clinical practice are concurrently advancing innovations in molecular optimization and strategy integration. Whether it is the emergence of new-generation ADC molecules or the exploration of “ADC+X” combination regimens, these efforts are continuously expanding the frontiers of ADC therapy. In the field of solid tumors, multi-target, multi-payload, and multi-modal combinations will help crack the code of resistance, whereas in the realm of autoimmune diseases, the exploration and application of customized solutions addressing immunogenicity and target complexity are expected to promote the precise clinical implementation of ADCs in non-malignant diseases.

## APPLICATION OF AI IN ADC DEVELOPMENT

The rapid development of AI technology is heralding a revolutionary transformation in ADC development. These computational methods can not only significantly accelerate development processes but, more importantly, have markedly improved design precision and success rates in existing studies. This will provide new solutions for addressing core scientific challenges in ADC development.

### AI-driven ADC molecular design

In the realm of monoclonal antibodies, AI technology is thoroughly transforming antibody design and optimization processes. This transformation is driven by AI’s capacity to learn the fundamental rules of antigen binding, to non-linearly optimize interdependent design parameters, and to generate novel antibodies *de novo*. Protein structure prediction tools like AlphaFold can accurately predict the three-dimensional structures of antibody-antigen complexes, guiding the optimization of antibody affinity and specificity^[162]^. Zheng *et al*. reported that antibody engineering guided by a deep generative model based on LSTM (Long Short-Term Memory) for structure prediction could increase affinity by up to 1800-fold, while AI-based final antibody generation and screening time could be reduced by 60%, concurrently minimizing off-target binding^[163]^. The latest version, AlphaFold 3, has further improved prediction accuracy, especially in forecasting protein-protein and protein-small molecule interactions. It can precisely identify key residues at binding interfaces, predict multiple potential epitopes on an antigen to support bispecific ADC design, and assess cross-reactivity risks^[164,165]^.

Concerning drug disposition and stability, molecular dynamics simulations combined with machine learning can predict the stability of different ADCs in blood circulation. A platform-based quantitative systems pharmacology (QSP) model developed by Scheuher *et al*. can predict the *in vivo* half-life of ADCs by considering factors such as pH, enzyme activity, and serum protein binding^[166]^. However, this method currently cannot provide direct guidance for the design of linkers and payloads.

Overall, while AlphaFold 3 builds upon the powerful protein structure prediction capabilities of AlphaFold 2 and further expands its application scope to accurately predict the interaction structures of complex molecules - including the binding modes of proteins with other proteins, ligands, RNA, and DNA - its application in prediction and development within the ADC field is currently limited to monoclonal antibody discovery. The simulation of more complex drug architectures like ADCs, which are composed of an antibody, a linker, and a drug, still awaits further research and exploration.

### AI in non-invasive prediction of receptor expression status

The therapeutic efficacy of ADCs is highly dependent on the expression level of the target antigen, and traditional detection methods require invasive biopsies to obtain tissue samples. This approach faces significant limitations in the assessment of neoadjuvant therapy or for metastatic lesions where tissue samples are difficult to obtain. Deep learning methods applied to digital imaging offer new possibilities for non-invasively predicting receptor expression status.

Digital pathology combined with deep learning has demonstrated immense potential in predicting receptor expression status. Results from the Phase I HEROHE challenge showed that HER2 status in breast cancer can be predicted solely from routine hematoxylin and eosin (H&E) stained slides, circumventing the need for special immunohistochemistry (IHC) techniques^[167]^. A deep learning model developed by Howard *et al*., integrating clinical features and digital pathology, achieved an area under the receiver operating characteristic curve (AUC) of 0.78-0.89 in external validation cohorts, significantly outperforming traditional clinical nomograms^[168]^. Research by Zhao *et al*. indicated that although some difficulties remain in assessing recurrence risk, molecular features of TNBC, including PIK3CA mutations, BRCA2 mutations, and PD-L1 expression, can now be predicted with considerable accuracy from whole slide images (AUC 95%CI > 0.65). This is highly valuable for guiding targeted ADC therapy and combination treatments^[169]^. This suggests considerable theoretical feasibility for such predictive applications in the context of ADC therapy, although research in this specific area remains nascent.

Deep learning-based prediction in medical imaging is also of significant importance. A deep learning model developed by Szep *et al*., based on whole-tumor apparent diffusion coefficient texture analysis, can predict the hormone status of breast cancer^[170]^. Although the term “ADC” in this specific study refers to a radiological parameter (Apparent Diffusion Coefficient) and not an ADC, the methodology it presents - using AI to analyze medical imaging for predicting tumor characteristics - is highly relevant for future ADC drug delivery and efficacy prediction. Radiomics methods developed by Chaunzwa *et al*. can predict the histological type and receptor expression of NSCLC from routine CT images^[171]^. Overall, such research holds considerable translational value in the ADC development field. However, these models are currently limited to specific diseases, and generalization to pan-cancer applications remains challenging. For the field of autoimmune diseases, model transferability is even more difficult, highlighting the challenges faced in using non-invasive prediction of receptor expression status in ADC development.

Moreover, translating these technologies into clinical practice also faces significant challenges. Harmon *et al*. pointed out that the generalizability of deep learning models across images acquired from different institutions and equipment is a key limitation^[172]^. Rai and Neri *et al*. emphasized that the “black box” nature of these models, i.e., their lack of interpretability, limits their clinical acceptance, necessitating explainable AI (XAI) techniques to enhance trust^[173,174]^.

In conclusion, while deep learning techniques for non-invasive prediction of receptor expression status hold broad prospects, their clinical translation still needs to overcome key challenges such as generalizability, interpretability, and prospective validation. With further technological advancements and in-depth clinical validation, these methods may eventually become important pillars of ADC precision therapy.

### Redefining the future of ADCs: AI, disease heterogeneity, and design innovation

Reflecting on the developmental trajectory of ADCs - from the materialization of the “magic bullet” concept to their widespread application in solid tumor therapy and tentative expansion into autoimmune diseases - we have witnessed the rapid evolution of this platform technology. However, with increasing depth in clinical and molecular understanding, a more fundamental reflection has emerged: Are we still employing 20th-century design logic to construct therapeutic systems for 21st-century complex diseases?

The traditional concept of ADCs is based on a linear “target-deliver-kill” logic, which is highly effective under conditions of high target expression, internalizable antigens, and acute treatment objectives. However, when ADCs are extended to chronic diseases characterized by high heterogeneity in target expression and requiring finely tuned effects (such as autoimmune diseases), this approach reveals its limitations.

Future ADCs may need to transcend the singular “antibody + toxin” structural paradigm and be re-envisioned as molecular delivery systems with adaptive regulatory capabilities. AI would no longer be merely a tool for molecular screening but would be embedded within the design logic, assisting in the construction of “intelligent ADCs” capable of real-time sensing and dynamic response: selectively releasing immunomodulatory factors in pro-inflammatory environments; maintaining a low-dose state during the repair phase; or even dynamically adjusting the payload release window based on the spatiotemporal expression of individual biomarkers. This concept not only addresses the discussions in this paper regarding “payload reconciliation” and “target heterogeneity” but also signifies the potential for a shift from structural-level optimization to system-level design.

As previously stated, autoimmune diseases do not always require the “elimination” of target cells but rather the “re-establishment of immune balance”. Therefore, future ADCs should perhaps be viewed more as “regulatory systems”, and their evaluation criteria should shift from toxicity intensity and killing efficiency to the ability to reconstruct immune homeostasis. This implies a fundamental shift in perspective: ADCs are no longer tools of confrontation but rather interveners for balance and repair.

After moving beyond the “structural engineering” phase of ADCs, we need to enter a phase of innovation in design logic. ADCs with true future potential will likely be AI-assisted adaptive systems possessing “dynamic decision-making capabilities”. Operating within this conceptual framework may be essential to overcoming the deep-seated obstacles currently faced in heterogeneous diseases, chronic treatment, and immune balance regulation, thereby advancing toward precision medicine in its truest sense.

## CONCLUSION

This review has systematically traced the technological evolution of ADCs since the proposal of the “magic bullet” concept, with a particular focus on elucidating the three key design elements - antibody, linker, and payload - and their synergistic roles in clinical translation. Benefiting from the targeting capability of high-affinity monoclonal antibodies and the “precision strike” mode of controlled payload release, ADCs have become important therapeutic options for hematological malignancies and various solid tumors, significantly broadening their range of indications. In areas such as autoimmune diseases, the CD30-targeting BV has brought initial clinical benefits for SSc, while studies on agents like ABBV-3373 have suggested the potential of ADCs to deliver immunomodulators and reduce systemic side effects in chronic immune diseases such as RA and SLE.

Nevertheless, the limitations of ADCs have also become increasingly apparent. Target antigen downregulation, alterations in endocytic pathways, or differences in microenvironmental enzyme activity can impair drug delivery efficiency; complex pharmacokinetics and cumulative toxicity add to the difficulty of long-term management; and immunogenicity and off-target effects warrant caution in autoimmune disease populations requiring lifelong treatment. Facing these challenges, next-generation ADCs are iterating toward site-specific conjugation, enzyme/acid/redox-sensitive linkers, and dual-target or dual-payload architectures to cover heterogeneous target cells and extend the therapeutic window. Furthermore, synergistic combinations with therapies such as ICIs and CAR-T cells are considered viable strategies for reshaping the immune microenvironment and enhancing antigen presentation efficiency.

Looking ahead, ADC design is transitioning from being “experience-driven” to “data-driven”. The integration of protein structure prediction tools with single-cell omics provides a powerful engine for rapidly screening targets and optimizing affinity and release kinetics; quantitative pharmacology models are also playing an increasingly important role in predicting circulatory stability and optimal dosing regimens. Driven by multi-center evidence-based research and industry-academia collaboration, ADCs are poised to further enhance their safety and efficacy, becoming a solid pillar in the field of precision therapy for cancer and autoimmune diseases, and offering new hope and tangible improvements in survival and quality of life for patients with refractory diseases.

## DECLARATIONS

### Copyright

© The Author(s) 2025.
